# Probing Limitations of Co-Alchemical Charge Changes in Free-Energy Calculations

**DOI:** 10.1021/acs.jctc.5c00192

**Published:** 2025-05-26

**Authors:** Nadine Grundschober, Dražen Petrov

**Affiliations:** 27270 BOKU University Institute of Molecular Modeling and Simulation, Department of Natural Sciences and Sustainable Resources Muthgasse 18, 1190 Vienna, Austria

## Abstract

Molecular dynamics simulations are nowadays one of the key methods to investigate the (thermo)­dynamics of protein–ligand binding at atomic resolution. The calculation of binding free energies of charged species is an encountered problem in molecular dynamic simulations. This is due to the approximation of the long-range electrostatic interaction. Here, we explore the discrepancies and biases of different approaches and whether and under which circumstances robust and reliable free-energy differences can be obtained using alchemical methods in combination with the lattice sum electrostatics treatment. Testing various setups and well-established approaches shows that the obtained free energies strongly depend on the initial setup choices. Different unconventional schemes, for example, placing more copies of perturbed species in a simulation box, were tested and showed partial success. Although they still suffer from pitfalls, they present a promising alternative approach in addressing the challenges related to non-neutral perturbations.

## Graphical abstract

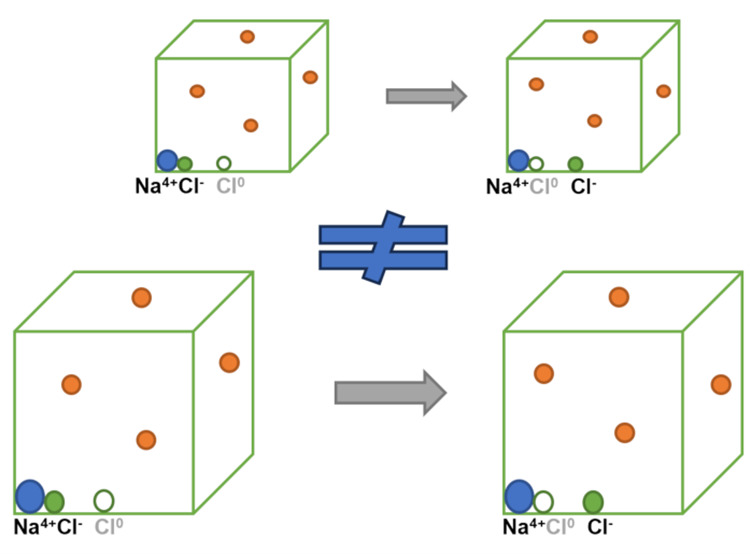

## Introduction

Molecular dynamics (MD) techniques are key tools for investigating and understanding the structure and dynamics of molecular systems. The chemical potential or the free energy is the driving force between all molecular processes and therefore an important quantity to characterize chemical and biological processes. For example, the strength of protein–protein interactions or the binding affinity of a ligand can be quantified by the calculation of free energies. One of the great challenges in MD simulations is the treatment of electrostatic interactions. Many biological systems of interest, including proteins, DNA, lipid molecules, ions, or the water environment, contain polar or highly charged species. Therefore, proper treatment of electrostatic interactions in MD simulations is important, since finite-size effects can lead to artifacts.^1–5^ This can result in the dependence of the calculated free-energy differences on system parameters, for example, the cutoff radius or the box shape and size.^6–9^

These interactions are typically handled via one or several approximation methods due to their high computational cost, since they only decrease slowly with increasing distance (1/*r*) between molecules.^10,11^ Two well established schemes are the reaction-field (RF) and the lattice-sum (LS), which both suffer from artifacts and bear an error when perturbations of non-negative net-charges are involved.^12–18^ Several correction schemes are available, such as post-simulation and instantaneous corrections. Using post-simulation charge-correction terms, based on the implicit-solvent Poisson–Boltzmann calculations, independence of system-related parameters can be achieved.^2,19–21^ Note that such corrections are applicable to both RF and LS methods and that they may also be applied instantaneously, although they are predominantly used in a post-simulation manner. This instantaneous correction scheme is based on the fact that the dominant errors depend on the total net-charge change of the system.^20^ In this case, the alchemical perturbation of the charged moiety is simultaneously performed with a counter-alchemical charge, i.e., perturbation of a remote molecule, usually a co-alchemical ion.^22–27^ It is worth mentioning that both of the above-mentioned approaches may still require additional corrections for the incorrect dielectric constant of a water model, the type of summation over the discrete water molecules, or the Galvani potential of moving the particles over the water–vacuum interface.^1,6^

Despite continuous research and improvements, calculating free-energy differences of charged species based on MD simulations remains challenging.^1,6,18,21,28^ Importantly, not only alchemical perturbation techniques, but also pathway methods as well as sampling of an equilibrium ensemble in plain MD simulations are affected.^18,29–31^ In a recent study, we have shown that such artifacts can be reduced or even removed when carefully considering the simulation setup, providing best practice recommendations.^31^ This study, however, has focused on species containing a single charge only.

The main goal of the present work was to explore the differences and biases of different free-energy approaches involving charged species when electrostatic interactions are treated by using the lattice sum approach. We ask whether and under what circumstances robust and reliable free-energy differences can be achieved. Different simulation setups focusing on ions bearing different amounts of integer net charges in combination with pathway and alchemical methods were tested. Furthermore, a charged protein–ligand system was used as an example of a practical use case. This study explores the limitations of free-energy calculations involving charged species and provides potential alternative approaches to address them.

## Results and Discussion

In this study, we investigate the impact of the simulation setup on charge-changing perturbation free energies, addressing potential biases introduced by charged species in combination with lattice-sum methods. To achieve accurate free-energy predictions comparable to experiment, in addition to the appropriate choice of the simulation setup, a number of factors such as sufficient sampling and force field accuracy play an important role. Moreover, charged species in combination with lattice-sum methods may introduce bias in conformational sampling, which, in turn, may also affect the free-energy calculation. To isolate the effects of the simulation setup and to eliminate or at least minimize any additional sources of bias, we primarily focus on a very simple two-ion *complex* system kept at an equilibrium distance from each other that represents a *bound* state. Here, we check for internal consistency under the assumption that a sufficiently large box would correspond to the correct result for a given force field. This approach allows us to investigate the influence of the simulation setup on the free-energy outcomes.

### Can Different Initial Choices Lead to Different Free Energy?

Alchemical perturbation involving charge changes may be performed simultaneously with another perturbation of an opposite net charge on a co-alchemical ion to ensure that the simulation box remains neutral throughout the simulation. This can be achieved using different simulation setups, e.g., different choices of the position or the total charge of the co-alchemical ion can be made. To test how such choices affect the calculated free energy we have constructed a simple test system in which two ions are kept in close proximity to each other with position restraints, representing a *bound* state, while an additional co-alchemical ion was restrained to a distant position from the *complex*. In particular, four different aspects were taken into account: (1) the total net charge of the nonperturbed ion (e.g., +1, +2, etc.), (2) the total charge of the coperturbed ion at the beginning of the perturbation (e.g., neutral or already charged), (3) the direction in which the coperturbed ion was displaced from the *complex* of two ions (e.g., along the *x*-axis or along the space diagonal of the simulation box), and (4) the distance to which the coperturbed ion was displaced (e.g., half the length of the box side (*a*/2) or half the length of the space diagonal of the box (*d*/2)). An example setup is shown in Figure 1, where the charge of the Cl^–^ in a *complex* with the Na^*n*+^ was perturbed to a co-alchemical Cl^0^ at the distance of *a*/2 in the *xyz*-direction while keeping the system neutral by adding free counterions. The details of all the setups are explained in the Methods section, and a summary of the simulated systems can be found in the Supporting Information Table S1.

**Figure 1:**
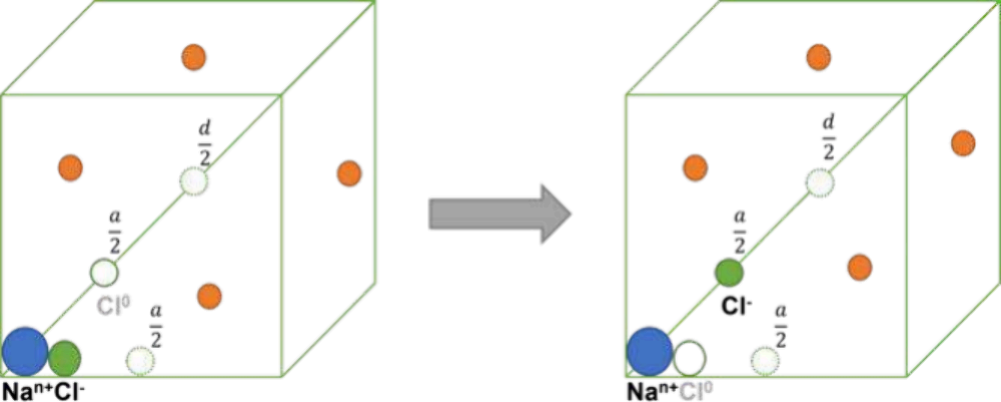
Graphical representation of an example perturbation in which a Cl^–^ ion in *complex* with a fixed charged Na^*n*+^ is neutralized and the neutral co-alchemically perturbed Cl^–^ ion at either (*a*/2) in the *x*- or *xyz*-direction or (*d*/2) in the *xyz*-direction is charged to −1 net charge. To ensure the overall charge neutrality of the system, *n* – 1 free counterions are added. Note that only one co-alchemical ion was used, and the other two green spheres represent alternative choices for the placement of the co-alchemical ion.

#### Perturbation Using a Charged Co-Alchemical Ion

The effect of the direction and the distance of the co-alchemical ion was examined by migrating the charge from the *bound* Cl^–^ to the co-alchemical ion in the *x*- or *xyz*-direction (see Figures S1 and S2). In this setup, the co-alchemical ion was assigned a charge at both end states to neutralize the system such that no free counterions were required. When in *complex* with a cation of a low charge (Na^+^), perturbations yielded the same free energy difference of approximately 36 kJ mol^–1^ regardless of the choice of the position of the co-alchemical ion (Figure S5, the Δ*G* values are listed in the Supporting Information Tables S2 and S3). The choice of the position of the co-alchemical ion starts affecting the outcome of performed free-energy calculations, with a striking difference of up to 15 kJ mol^–1^ when comparing, for instance, the displacement of (*a*/2) in the *x*- and (*d*/2) in the *xyz*-direction for the Na^4+^ system. However, with higher charges, the uncertainty in free-energy calculations also increases, as depicted in large error bars (Figure 2), impacting the comparison of ΔΔ*G* between the setups.

**Figure 2:**
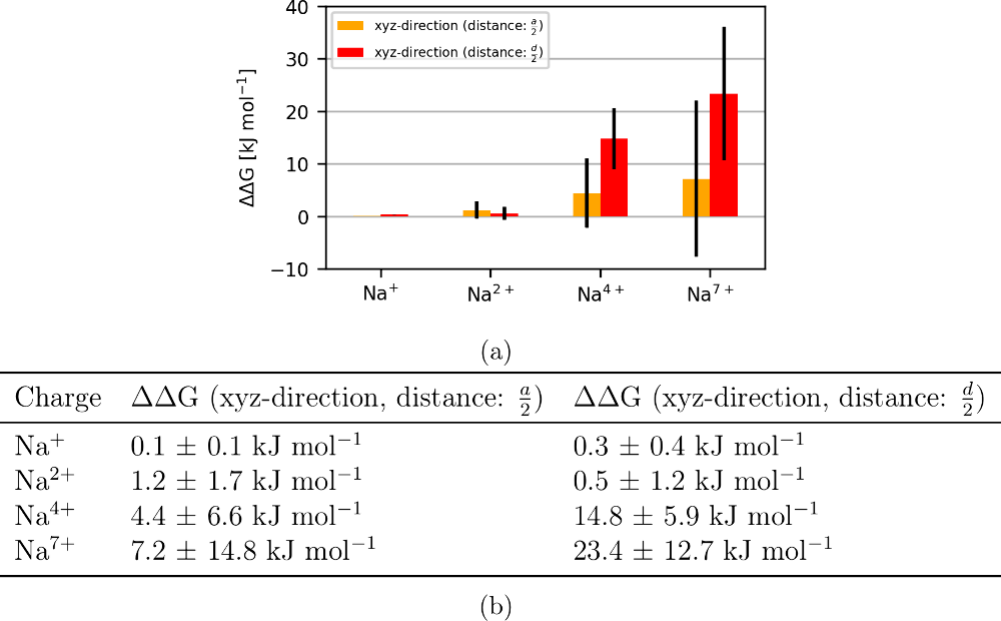
(a) Bar graph and (b) table are showing the difference between the free energies of the displacement of the co-alchemical ion dependent on the direction or the distance, normalized to the free energy calculated when the displacement in *x*-direction was used. Different choices of displacement of the co-alchemical ion lead to different free-energy differences, with increasing charge on the Na^*n*+^ ion. This, however, also coincides with large error estimates.

Such a noisy data are potentially caused by a large charge on the co-alchemical ion (Cl^(*n*–1)–^ and Cl^*n*–^ in the two end states) that is concentrated in one particle, which may lead to artifacts as such a particle does not correspond to any physical ion. This setup also resulted in very large free-energy differences, which altogether suggest that such a setup is not suitable for robust calculation of free-energy changes upon co-alchemical perturbations and therefore should be avoided in cases of larger total net charge of the system of interest. In a next step, the same *bound* states were considered using a neutral co-alchemical ion with free counterions.

#### Systems Neutralized with Free Counterions

The charge of the *in-complex* Cl^–^ was migrated to the coperturbed ion (neutral at the beginning of the perturbation), which was placed to the same distance (*a*/2) in either the *x*- or *xyz*-direction or to (*d*/2) in the *xyz*-direction (see Figures S3 and S4). To keep the systems neutralized, *n* – 1 free Cl^–^ counterions were used in combination with Na^*n*+^ in the *complex*. Figure 3 shows that even with a larger charge of the Na^+^ ion, the direction and the distance of the coperturbed ion has almost no influence on the obtained free energies. Only with large charges do the direction and the distance start to play a role. Up to a charge of Na^7+^, the results are independent of the direction (when using the same distance of *a*/2, with a difference within 0.5 kJ mol^–1^). For the setup Na^7+^, the difference between the free energies when the co-alchemical ion is displaced in the *x*- and *xyz*-directions (same distance) is 13.5 ± 2.1 kJ mol^–1^ and 19.7 ± 2.3 kJ mol^–1^, respectively, when the distance of displacement is increased to (*d*/2). The setups with Na^4+^ show a discrepancy of 3.1 ± 2.1 kJ mol^–1^ when different distances and directions of displacement for the co-alchemical ion are used. Furthermore, the error estimates are much smaller compared to the previous setup using a charged co-alchemical ion, suggesting that this type of setup strategy may be the preferred one.

**Figure 3:**
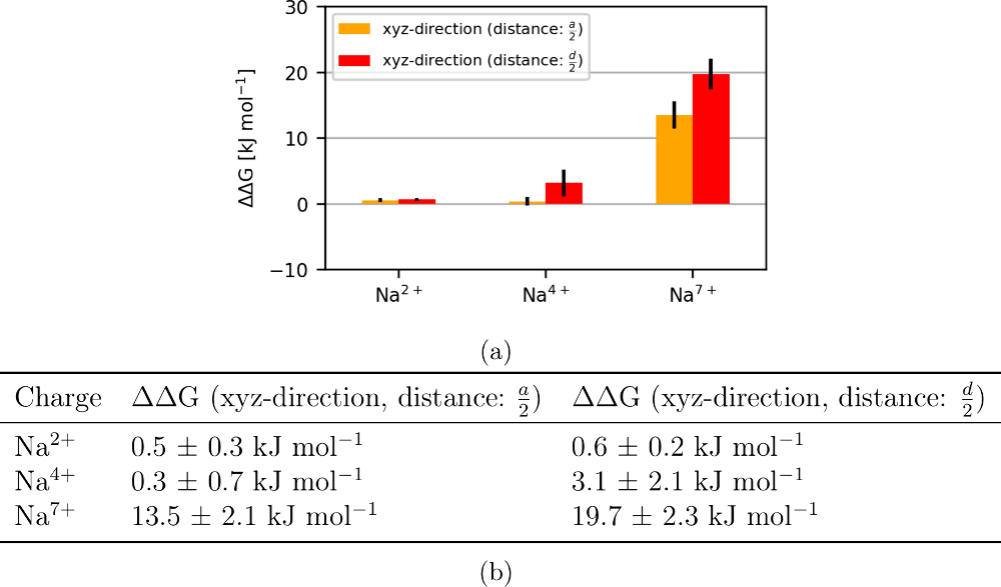
(a) Bar graph and (b) table are showing the ΔΔ*G* values of the two directions and distances, normalized to the calculated free energy when the displacement in the *x*-direction was used. With an increase of the charge, the position and distance of the perturbed ion affects the calculated free energy. The ΔΔ*G* values independent of the direction or distance are very small up to a charge of Na^2+^. Using a charge of Na^7+^ results in a big difference (13.5 kJ mol^–1^) between these two directions, and the distance of the perturbed ion starts playing a role.

These results show, in accordance to previous studies, that robust free energy estimates independent of the exact setup choices of co-alchemical perturbations may be obtained when the net charge of individual species is small.^2,18,31^ However, this is not the case for larger net charges, e.g., Na^4+^ (the Δ*G* values are listed in the Supporting Information Tables S4 and S5). While the Na^*n*+^Cl^–^ *complex* is an nonphysical construct, it serves as a simple test system to examine methodological limitations. For example, many proteins or their binding pockets bear a large net charge, suggesting that perturbation free-energy calculations of charged ligands bound to such proteins may suffer from the same pitfalls.

We further tested whether this still holds when umbrella sampling is employed. Similarly to the perturbation approach, calculated free energies diverge when a larger net charge of Na^4+^ is used and the Cl^–^ is pulled in the *x*- or *xyz*-direction (Supporting Information Table S6). For a charge of Na^4+^, the PMF is similar across different directions, but the Δ*G* values increase between box sizes of 4 and 8 nm when pulling the co-alchemical ion in both *x*- and *xyz*-directions (Figure S7). Overall, the deviation between various setups in many cases exceeded 1 k*T* (at 300 K), showing that using simple (conventional) co-alchemical perturbation or umbrella sampling may carry artifacts. Therefore, alternative setups were employed, for instance, by using larger simulation boxes.

### The Role of the Box Size

In the next step, different sizes of the simulation box were used to test their influence on the calculated free energies. The Na^+^ ion was charged with +2 and +4, and the charge of the Cl^–^ was migrated to a coperturbed ion (neutral at the beginning of the perturbation) at (*d*/2) in the *xyz*-direction, while the box was neutralized with free counterions.

Figure 4 shows that with a charge of +2, the box size does not have an impact on the calculation. Increasing the charge of the Na^+^ ion to +4, the free-energy differences increased from 329.8 ± 2.0 kJ mol^–1^ in a 4 nm box to 337.3 ± 0.7 kJ mol^–1^ in an 8 nm box.

**Figure 4:**
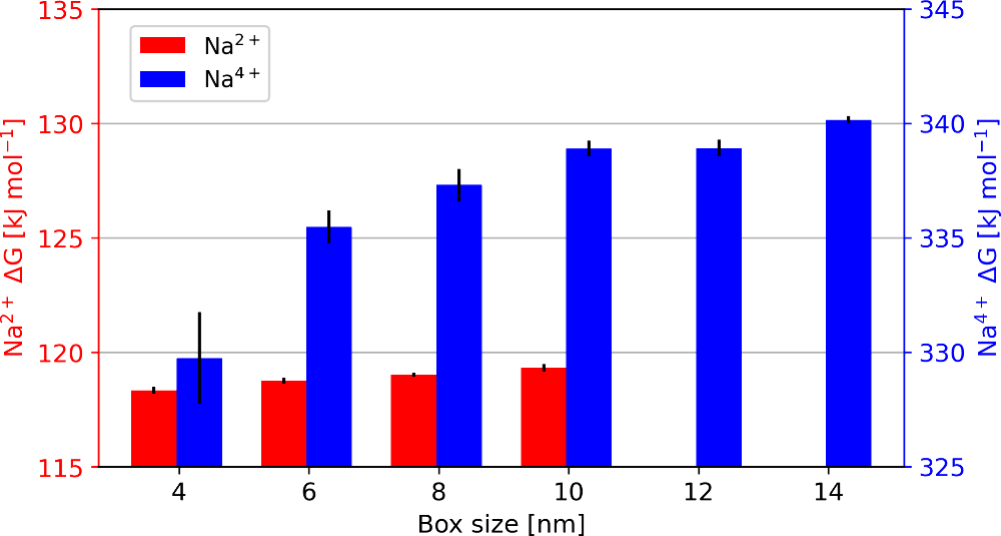
Charge of the Cl^–^ ion of the Na^2+^Cl^–^ and the Na^4+^Cl^–^ *complex* was migrated to *d*/2 of the box length side in a 4, 6, 8, 10, 12, and 14 nm box. For systems containing small charges, the box size does not matter, whereas, for the systems with a charge of Na^4+^ the calculated free energies are strongly dependent on the box size.

This suggests that for the systems with a charge of Na^2+^, free energies independent of the box size can be achieved. This is also consistent with the tests on the direction and the distance of displacement of the co-alchemical ion (Figure S6). However, with a larger charge, the calculated free energies depend on the size of the box. For example, the difference using the Na^4+^ between a 4 and a 10 nm box are 3.7 k*T* (at 300 K), which suggests the need to perform simulations in big boxes (Table S7). Additionally, for the Na^4+^ setup a box size of at least 10 nm is necessary to obtain free energies, which are not influenced by the box size (Figure 4). Another possibility might be the application of post-simulation corrections or a correction factor.

### Reversed Two-Ion *Complex*

In this setup, the positively charged ion (Na^+^), in *complex* with a Cl^2–^ ion and a Cl^4–^ ion, was coperturbed with another Na ion (neutral in the initial state) that was position-restrained at a distance of *d*/2 (displaced in the *xyz*-direction) from the *complex*. The dependency of the free energies on the box size was examined by using six different box sizes.

The results imply that the free energy with a charge of Cl^4–^ depends on the box size and increases with increasing box size (Figure 5 and Table S8). This trend plateaued only at a box size of 12 nm side length, with values of 351.1 ± 3.1, 344.6 ± 6.2 and 352.2 ± 4.5 kJ mol^–1^ for box sizes of 12, 14, and 16 nm, respectively. Strikingly, the difference between the free energies obtained in a large (14 nm) and a small (4 nm) box is approximately 100 kJ mol^–1^, which is much larger than that for the Na^4+^ setup. In this case, the difference between the free energies calculated in a 4 nm box and those calculated in a 10 nm box is 10× smaller. Free-energy calculations with Cl^2–^ suffer from the same problem, yielding a difference of 10 kJ mol^–1^ between the smallest and the largest box, which was not observed for the Na^2+^ case (Table S7 and Table S8).

**Figure 5:**
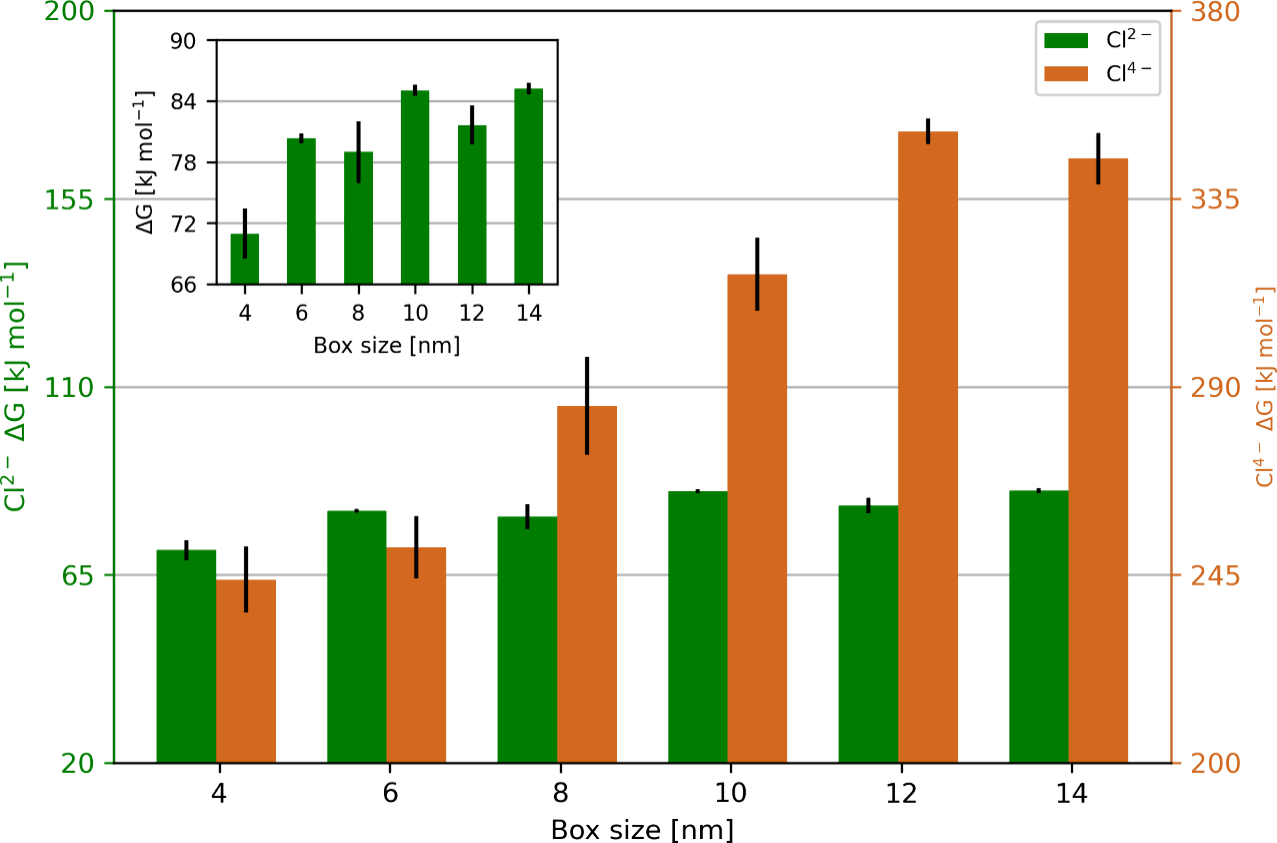
The charge of the Na^+^ ion of the Cl^2–^Na^+^ and Cl^4–^Na^+^ complexes was perturbed in *xyz*-direction. The results of both setups suggest a dependency of the free energies on the box size, which is more pronounced in the Cl^4–^Na^+^ complex. The inset shows a zoomed-in view of the results for Cl^2–^.

#### Effect of Salt Concentration on the Free Energy Box-Size Dependence

As previous studies suggest that the addition of salt may reduce finite-size electrostatic artifacts,^31,32^ we tested the effect of a physiological salt concentration of 0.15 M NaCl in this setup. Here we focused on three different *complexes* (Na^4+^Cl^–^, Cl^2–^Na^+^, and Cl^4–^Na^+^) that showed a box-size dependence in free-energy calculations without salt. Similar box-size dependence to simulations without salt was observed (Figure 6 and Table S9) for the Na^4+^Cl^–^ *complex*, with more than 10 kJ mol^–1^ difference between the smallest and the largest box. On the other hand, the discrepancy in obtained free energies in different box sizes for the Cl^2–^Na^+^ and Cl^4–^Na^+^ systems was largely reduced compared to the no-salt systems. For example, the difference between the smallest and the largest boxes for the Cl^4–^Na^+^ system is approximately 10 kJ mol^–1^ with salt, compared to more than 100 kJ mol^–1^ when no salt was added. Additionally, the difference to the free energy calculated in the largest simulation box used (14 nm) decreases much faster with the box size in simulations with salt. This suggests, in agreement with our recent study,^31^ that the addition of salt reduced the extent of the box size dependency.

**Figure 6:**
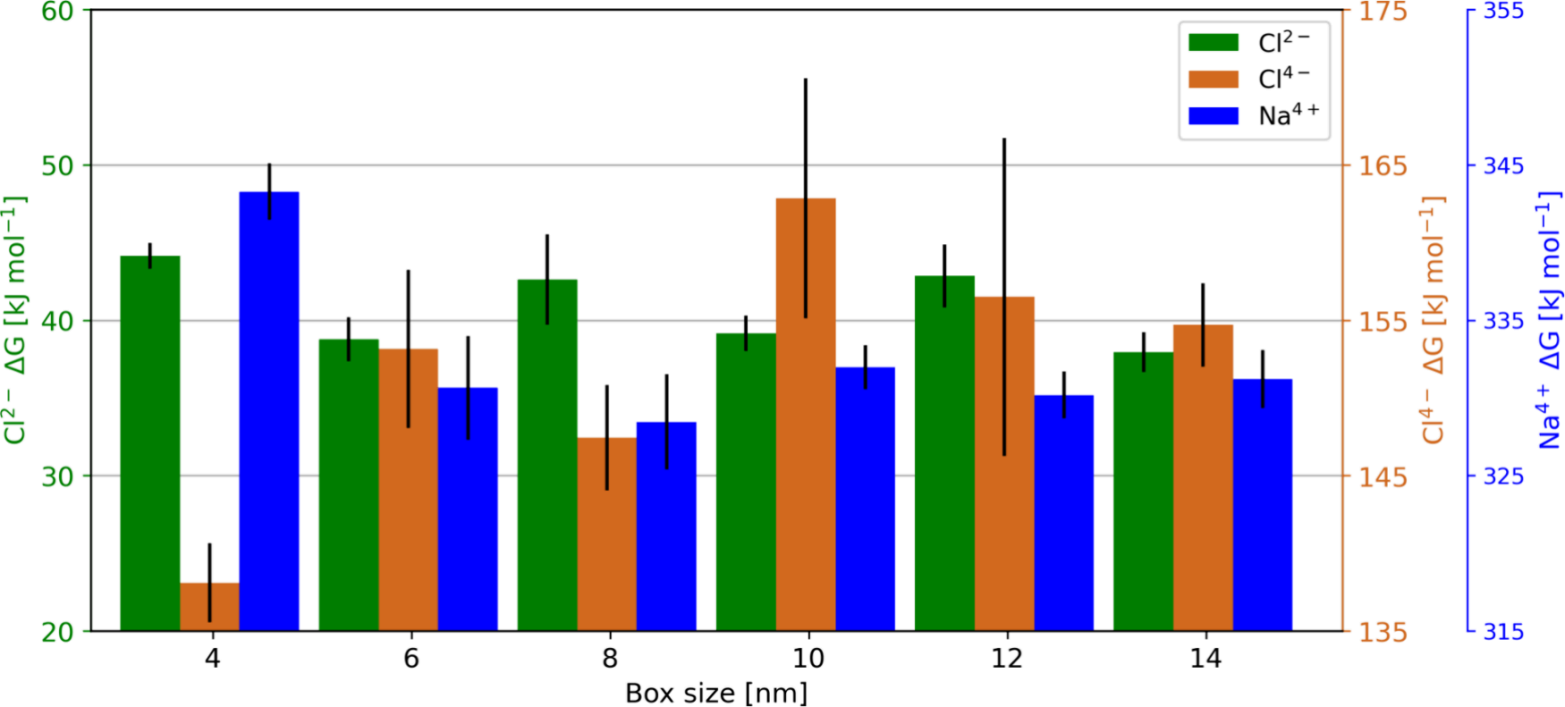
In three different setups (Na^4+^Cl^–^, Cl^2–^Na^+^, and Cl^4–^Na^+^), a physiological salt concentration of 0.15 M NaCl was added to the systems. The results show the largest effect of the box size between 4 and 6 nm in all setups. Overall, the box-size dependency of Δ*G* values also persists in this setup, albeit to a smaller extent.

### Effect of Co-Alchemical Charge Changes on a Protein–Ligand System

A charged protein–ligand system was used to assess the effect of a co-alchemical charge change as an example of a practical use case. Here we use the protein Hif2α that carries a net charge of −5 and one of its known binders (ligand-290) with a net charge of +1. The charge of the amino group of the ligand-290 in the active site was neutralized and coperturbed to a co-alchemical ion, which was kept at a distance of half the box diagonal along the space diagonal of the box. The calculated free energies in a small 6 nm box are roughly 20 kJ mol^–1^ greater compared to those in an 8 nm box, regardless of whether the simulations were performed with or without ions. Interestingly, the calculated free energy increases to a similar value obtained in the 6 nm box, with a box size of 10 nm (Figure 7). When larger boxes are used, the free energy again decreases and reaches values of −74.2 ± 5.5 kJ mol^–1^ and −87.0 ± 3.4 kJ mol^–1^ in simulations using a 14 nm box size, without and with salt, respectively (Table S10). The former value (from simulations without salt) shows a larger deviation from the obtained free energy in the smallest 6 nm box. In addition to the direct effect of electrostatic finite-size artifacts on the calculated free energy, it is worth noting that they also may bias configurational sampling for this system,^31,32^ which would in turn cause further discrepancies in the calculated free energies.

**Figure 7:**
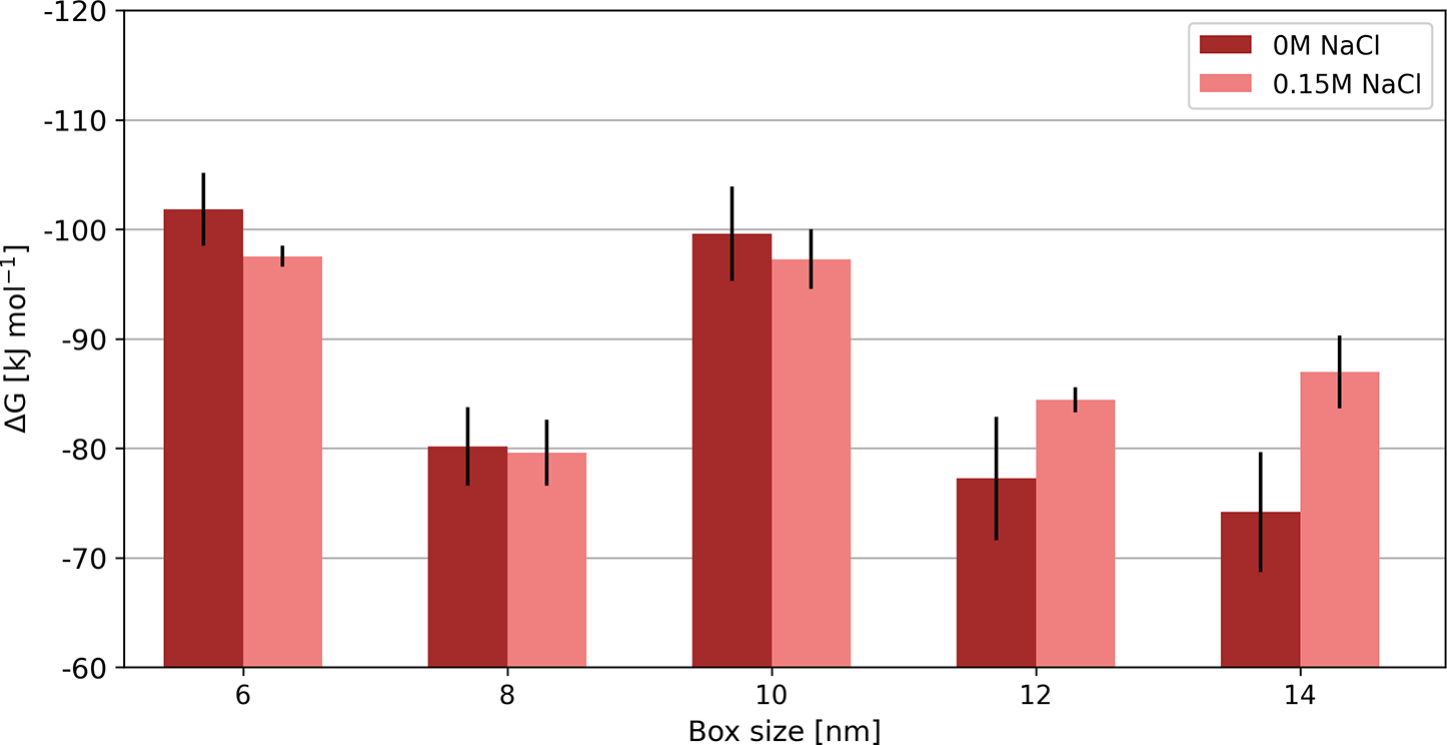
Free-energy differences of neutralizing the charge of ligand-290 in the protein Hif2α. Calculated free energies show box-size dependence, where this effect is more pronounced for simulations without salt. Additionally, a similar nonsystematic effect is observed at the box size of 8 nm for both setups.

### Alternative Approach

Using typical setups with larger charges and different configurations resulted in free-energy differences dependent on the exact simulation choices, for example, on the placement on the co-alchemical ion or the box size. Thus, alternative approaches were tested.

#### Bigger Box with Multiple Copies of the *Complex*

The results above showed that a large box (side of at least 10 nm) is required to obtain free energies, which are independent of the box size, when the *complex* consists of species with increasing net charge (e.g., +4) and no additional salt concentration is used. Since performing MD simulations is computationally expensive, especially for large simulation boxes, the *available* space can be used by putting more copies of the *complex* and the corresponding co-alchemical ion in larger boxes. The main idea is to use more copies as independent free-energy calculations, which would allow for better statistics with no additional computational cost of running independent simulations. To check if this assumption holds, i.e., that the copies within one box can be treated as independent, this setup was first tested with a neutral compound, the asparagine side chain analogue. Four copies of the asparagine side chain analogue were placed in a 6.35 nm box and perturbed into a neutral noninteracting dummy compound. Placing a single copy of four copies in a box resulted in similar total free energy differences per copy compared to a single copy (see Table S11).

The setup Na^+^ and Na^4+^ with free counterions was used, and three, four, or eight copies were placed in a box. For the 4 and 6.35 nm boxes four copies were put in the box, and for the 8 nm box eight copies were put in the box. Initial coordinates are listed in the Tables S12a, S12b, S13a, and S13b. After the MD simulations, in addition to the free energy of the complete multicopy perturbation, the free energy of perturbing the individual copies was calculated by reanalyzing the collected trajectories, where only one copy was perturbed and others were kept in a charge-neutral state (Δ*G* values are listed in the Supporting Information Table S14).

The four copies of the Na^4+^ setup in the 4 and 6.35 nm boxes result in similar free energies for each copy, whereas the free energies of the individual copies in the 8 nm box differ (Figure 8). Moreover, consistent with the Na^4+^ setup, the normalized Δ*G*_mult_ per copy is lower compared to the disentanglement approach, where the free energy for each individual copy is obtained in postprocessing analysis assuming other copies remain unperturbed (see the Methods section for details, Figure S8). The calculated values of Δ*G*_mult_/copy in boxes of different sizes show similar values, all underestimating the free energy compared to the reference Δ*G*_single_. On the other hand, the average over the disentangled free energies of the copies overestimates the free energy compared to the reference, with the exception of the setups when different orientations for displacing the co-alchemical ions were used. Notably, this approach yields a correct free-energy estimate when 3 copies are placed in a 6 nm box and even with 4 copies in a 6.35 nm box (which corresponds to a density similar to a single copy in a 4 nm box). This showcases the importance of the choice of the position of the co-alchemical ions. More importantly, the results suggest that disentanglement of multiple copies of perturbed species is possible and that free energies comparable to the reference may be obtained when varying orientations for displacing of the co-alchemical ions is used. However, further investigation is required to develop a more robust method for perturbing multiple copies simultaneously and for the related disentanglement of free energies.

**Figure 8:**
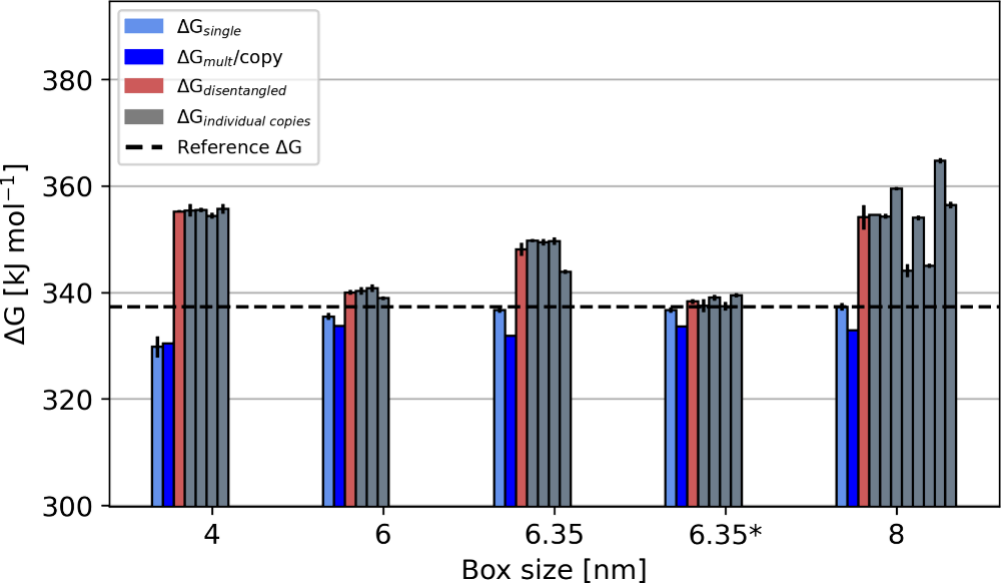
The graph shows a comparison of different Δ*G* values representing: the Δ*G* of a single copy in the box in light blue (Δ*G*_single_, data from Figure 5), the total Δ*G* of multicopy perturbation normalized by the number of copies in dark blue (Δ*G*_mult_/copy), the average Δ*G* of the disentangled copies in the box in red (Δ*G*_disentangled_) and the disentangled Δ*G* values of the individual copies using the setup Na^4+^. Furthermore, the free energies are compared to the reference Δ*G*, the Δ*G* of a single copy in the largest simulated box (Figure 4). Interestingly, the disentanglement of the individual copies results in higher free energies compared with a single copy or the total free energy per copy. In the boxes marked with an asterisk (*), the copies were oriented differently, which resulted in Δ*G* values comparable to the reference Δ*G*_single_.

Since the orientation of the displacement of the co-alchemical ion matters, an attempt was made to dislocate the charge in several directions around the Na^4+^Cl^–^ *complex*. A setup in which the migrating charge is distributed across six coperturbed ions was tested. These ions were displaced in the *x*-, *y*-, and *z*-directions around the Na^4+^Cl^–^ *complex* using five different box sizes. In this setup, however, free energies dependent on the box size were obtained (Supporting Information Figure S9, Table S15, and Table S16).

## Conclusions

In this article, not only different variations of a standard co-alchemical perturbation setup but also alternative setups were applied to test the influence of different charged particles on the obtained free energies when the lattice sum approximation to electrostatic interactions was applied. In systems with small charges (up to Na^2+^), the direction and the distance of the displacement of the co-alchemical ion have little impact on the obtained free energies. With an increase of the charge, the choice of certain configurations, such as the amount of the perturbed charge or the distance, becomes important. We have shown that this holds true for the umbrella sampling approach as well.

Using different box sizes showed that a box side length of at least 10 nm is required for a setup with the Na^4+^Cl^–^ *complex* to obtain box-independent free energies when no additional salt concentration is used. This effect is even more pronounced when perturbing the positive charge, where the Cl^4–^Na^+^ *complex* required a 12 nm box, resulting in a free-energy difference of more than 100 kJ mol^–1^ in contrast to a small 4 nm box, which is 10-fold greater compared to the setup perturbing the negative charge. Additionally, this artifact persists for a Cl^2–^Na^+^ *complex*, albeit to a smaller extent, in contrast to a Na^2+^Cl^–^ system that yields box-size-independent free energies. The observed box-size dependence persists when a salt concentration of 0.15 M NaCl is added to the simulation box, although to the reduced extent, especially in the case of the Cl^4–^Na^+^ *complex*. Futhermore, charging free energies in a protein–ligand system, with a net charge of −5 for the protein and a +1 charge for the ligand, also shows box-size dependency, with the largest difference in the obtained values between different box sizes of approximately 20 kJ mol^–1^ in simulations both with and without salt concentration.

This suggests that simulation boxes much larger than typical ones are required to obtain reliable free energies when simulating systems containing molecules of larger net charge, particularly if no additional salt concentration is used. To use the available volume in such boxes and to reduce overall computational costs, more copies of perturbed species can be simulated simultaneously in the same simulation box. This, however, leads to the underestimation of the free energy if a simple average per perturbed copy is calculated compared to the reference value from a single copy in a big box. In addition, free energies of perturbing individual copies can be obtained in a post-simulation disentanglement analysis, which overestimates the free energy compared to the reference. However, when the co-alchemical ions of individual copies are displaced in different directions, comparable free-energy estimates to the reference values (largest simulated box) are achieved. This suggests that disentangling perturbation free-energies of individual copies is a promising approach in addressing the box-size dependence of free-energy calculations and related computational costs when large boxes are required.

Applying a similar idea of using different directions for co-alchemical ion displacement in a system containing a single perturbation species, by distributing the perturbed charge around the *complex*, leads to a similar outcome as the *simple* setup with box-size dependent free energies. This shows that splitting up the perturbed charge and distributing it over a multiple co-alchemical ions is not a suitable approach for addressing this issue.

Summing up, depending on the size and the sign of the charge of the species in the system, free energies calculated using a typical coperturbation setup may strongly depend on the initial choices, the box size in particular. The usage of a neutral co-alchemical ion at the beginning of the perturbation and additional a salt concentration helps alleviate the observed artifacts, however, not always resulting in their complete removal. One approach to address the artifacts might be to test different box sizes to obtain box-size independent free energies; however, this requires more computational time. Using the available space in a large simulation box by placing more copies of perturbed species and treating them independently proves to be a challenging yet promising alternative approach.

## Methods

### Simulation Setup

MD simulations were performed using the GROMACS simulation package,^33–35^ version 2020, with a 2 fs integration step. The united-atom GROMOS force field parameter set 54A8,^36^ together with SPC water model,^37^ was used. Long-range electrostatic forces were taken into account by means of the Particle–Particle-Particle-Mesh algorithm with an analytical derivative for long-range electrostatic interactions^38^ while keeping the simulated systems neutral. The Verlet pair-list algorithm^39,40^ with van der Waals and Coulomb interactions was truncated to 1.4 nm. The temperature and pressure in the systems were controlled using weak coupling^41^ with a relaxation time of 0.1 ps and kept constant at 300 K and 1 bar. Pressure scaling was applied isotropically, with an isothermal compressibility of 4.5 × 10^–5^ (kJ mol^–1^ nm^–3^)^−1^. Initial velocities were assigned according to the Maxwell–Boltzmann distribution, and the systems were equilibrated for 200 ps at 300 K. The coordinates were saved every 10 ps and the energies every 2 ps. Three independent simulations were performed for all systems.

Twenty-one (equidistant λ-points) simulations were performed along the alchemical path between the two physical states. The MD simulations were performed for 5 ns for each λ-point. If not stated otherwise, only charge perturbation was performed, while keeping the Lennard-Jones parameters intact.

The MD trajectories were analyzed by using GROMACS analysis programs, e.g. gmx bar or gmx wham, and the free-energy differences were calculated using the multistate Bennett acceptance ratio (MBAR) using the Python packages SMArt^42^ and pymbar.^43^ The statistical errors were estimated with bootstrapping, by randomly resampling the energy trajectories 100 times.^42^ The visualization of the setup was performed with the PyMol program^44^ and the Python packages matplotlib^45^ and pandas,^46^ version 1.1.3, was used to create the graphs.

Additionally, the *bound* state was defined such that the position of Na^*n*+^ was always restrained at the coordinates (0, 0, 0) and at (0.27, 0, 0) for Cl^–^ with a force constant of 1,000,000 kJ mol^–1^ nm^–2^ in all directions. The co-alchemical ion was position-restrained at a distance of half the box size length (*a*/2) or half the box diagonal (*d*/2) in the *x*- or *xyz*-direction using the same force constant as for the two ions in the *bound* state.

### Free-Energy Calculations Applying Alchemical Methods

The free-energy difference of transferring the charge from an ion in a *complex* with another ion of opposite charge to a co-alchemical ion was calculated. A simple setup consisting of one positively charged ion (Na^*n*+^) and one negatively charged ion (Cl^–^) was tested. The charge of Na^*n*+^ was kept unperturbed, whereas the charge of Cl^–^ was perturbed to Cl^0^ in all setups. To ensure overall charge neutrality of the simulation box, another anion, placed at a half box length in the *x*- or *xyz*-direction ((*a*/2, 0, 0) or (*a*/2, *a*/2, *a*/2), respectively, where *a* is the length of the box side), was coperturbed.

The influence of various simulation setups on the free-energy calculation was tested, including, for instance, the charge of the ions, the placement of the second ion used for co-alchemical perturbation, the box size, and the addition of salt. All setups are described in more detail below.

#### Charge and Configuration

##### Charge of Ions

Within the *bound* state, the Na cation was charged a certain amount (Na^*n+*^), and a single charge of the Cl^–^ was perturbed and migrated to the co-alchemically perturbed ion. Two different setups related to the charge of the coperturbed ion were applied: (1) starting with a negatively charged Cl^(*n*–1)–^ and perturbing it to Cl^*n*–^ and (2) starting with a charge neutral ion Cl^0^ and charging it to a Cl^–^, with (*n* – 1) additional free counterions (Cl^–^) keeping the system neutral. A summary of the used setups is shown in Table 1 and Table S1, starting with a single charged Na ion. The charge of the Na ion was subsequently increased, reaching Na^7+^ as a maximum to also test an extreme. The two ions forming the *bound* state and the co-alchemical ion were position-restrained, with other counterions moving freely. These setups were tested in a 4 nm box applying co-alchemical perturbation in *x*- and *xyz*-direction.

**Table 1:** Overview of the Setups Regarding the Different Charge of Na^*n*+^ and the Addition of Free Counterions

charge	without free counterions	with free counterions
Na^+^	√	
Na^2+^	√	√
Na^4+^	√	√
Na^7+^	√	√

##### Displacement of the Coperturbed Ion in the *x*-direction

Figures S1 and S3 show the positions of the ions, with the two-ion *complex* in the origin and the coperturbed ion displaced along the *x*-coordinate. In both schemes the charge was shifted to the coperturbed ion, which is placed at a half of the box length in *x*-direction (*a*/2, 0, 0). In Figure S1, the setup with free counterions is displayed, whereas in Figure S3 the coperturbed Cl^–^ ion is charged and no free counterions are required.

##### Displacement of the Coperturbed Ion in the *xyz*-direction

A similar setup was used in this setup, where the co-alchemical ion was displaced in the *xyz*-direction, i.e., along the space diagonal (Figure S2b and Figure S4b). In order to test both the direction and the distance to which the coperturbed ions was displaced, the charge was shifted to the coperturbed Cl^–^ ion placed at half of the length of the diagonal (*d*/2) of the box, which corresponds to (*a*/2, *a*/2, *a*/2), or at half of the box size length (*a*/2) along the diagonal, which corresponds to $\left(\frac{a\sqrt{3}}{6},\frac{a\sqrt{3}}{6},\frac{a\sqrt{3}}{6}\right)$ , shown in Figure S2a and Figure S4a.

##### Box Size

Since with a larger charge the distance between the *complex* and the coperturbed Cl^–^ ion seems to matter, larger box sizes, and therefore greater distances, were tested. The setups consisted of three different charged Na^+^ ions and six different box sizes (Table 2). The box size starting at 4 nm was increased in 2 nm steps until free energies independent of the box size could be achieved.

**Table 2:** ^a^

	box size [nm]					
charge	4	6	8	10	12	14
Na^2+^	√	√	√	√		
Na^4+^	√	√	√	√	√	√

a The Na^2+^Cl^–^ *complex* was placed in four different box sizes, whereas the Na^4+^Cl^–^ *complex* was put in six different box sizes.

The setup with additional free counterions keeping an overall neutral charge was used. The perturbation was performed in the *xyz*-direction, and the Cl^–^ ion was placed at (*d*/2) of the box.

##### Reversed Two-Ion *Complex*

In order to verify if the same conclusions can be drawn when the positive charge is perturbed, a *complex* of a Cl anion with a charge of −2 and −4 and the Na cation with a charge of +1, together with a co-alchemically perturbed Na cation (from Na^0^ to Na^+^) displaced in *xyz*-direction, was simulated. The total charge of the box was neutralized by the addition of one and three free Na^+^ counterions, respectively. The following box sizes were tested: 4, 6, 8, 10, 12, and 14 nm.

##### Protein–Ligand System

The crystal structure of Hif2α (total net charge of −5) was downloaded from the protein data bank, PDB code 5TBM, and the ligand-290 ^47^ was placed in the active site. The united-atom GROMOS force field parameter set 54A8,^36^ together with SPC water model,^37^ was used. The parameters of the MD simulation were as described above, except that the co-alchemical ion was distance restrained in the *x*-, *y*-, and *z*-directions at the distance of half the box length from the perturbed amino group using a force constant of 10,000 kJ mol^–1^ nm^–2^. The net charge of +1 of the amino group of the ligand was neutralized while simultaneously perturbing the charge to the co-alchemical ion. Five different box sizes without and with a salt concentration of 0.15 M NaCl were simulated. The same free-energy calculation protocol was used as for the two-ion *complexes*.

#### Alternative Approaches

##### Copies

To use the available space in the box and to collect data of multiple replicates in a single simulation, more copies of the two-ion *complex* and the coperturbed ion were put in a simulation box. Different setups were tested to explore and evaluate several options. First, a straightforward approach was used, putting four copies of the two-ion *complex* with their respective co-alchemical ion displaced in the *x*-direction at a distance of half the box length. The copies of the two-ion *complex* were placed at the origin and at the center of the 3 adjacent box sides (e.g., at (*a*/2, *a*/2, 0)), all placed in a 4 and 6.35 nm box (Figure 9a). Similarly, eight copies were placed in a 8 nm box, where the copies of the two-ion *complex* were placed such that they form a cube of (*a*/2) side (i.e., the first one in the origin and the last one at (*a*/2, *a*/2, *a*/2)), in the origin and the others and the matching coperturbed ion were placed at (*a*/4, *a*/4, *a*/4) from the *complex* (Figure 9c). Two different charge setups, Na^+^ and Na^4+^, were tested. Three copies were put in a 6 nm box, and each of the coperturbed ions was moved (*a*/2) in either the *x*-, *y*- or *z*-direction from its respective *complex* (Figure 9d, Table S13a). Note that 4 copies in a 6.35 nm box and 8 copies in an 8 nm box present the same concentration as 1 copy in a 4 nm box. In this case the Na^4+^ charge setup was used. Details of the initial coordinates are provided in Tables S12a, S12b, S13b, and S13a.

**Figure 9:**
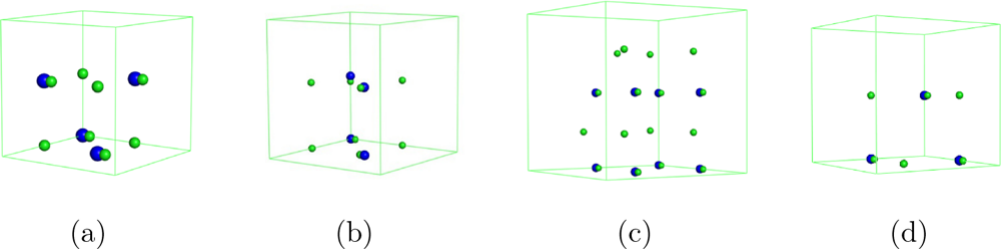
Overview of the position of the copies in each setup. (a) Setup of four copies of the two-ion *complex* and the coperturbed ion in a 4 and 6.35 nm box. All copies of the two-ion *complex* were oriented in the *x*-direction. (b) The orientation of the two-ion *complex* is different for each copy placed in a 6.35 nm box. Furthermore, the coperturbed ion of two copies is moved in the opposite direction compared to the other two copies. (c) Eight copies were placed in the 8 nm box. The coperturbed ion was positioned at (*a*/4, *a*/4, *a*/4) from the initial position. (d) All six directions were covered by putting three copies in a 6 nm box and the coperturbed ion was moved to (*a*/2) of the box length side.

##### Disentanglement

The free-energy difference per single copy in the simulated box was calculated by simply dividing the total free-energy change by the number of copies. An alternative approach to disentangle the copies into individual perturbations and recalculate the free energy with the same trajectory using a set of new topologies was applied. In each of these new topologies, one copy (one two-ion *complex* with associated coperturbed ion) is perturbed individually whereas all the other copies remain frozen in their initial state.

## Supplementary Material



## Notes

Description and representation of individual simulation setups with initial positions of all relevant particles, together with the obtained free-energy differences (PDF)
