# Supplementary material for: Probing Limitations of Co-Alchemical Charge Changes in Free-Energy Calculations
Source: J Chem Theory Comput. 2025 May 26;21(11):5669–78. doi: 10.1021/acs.jctc.5c00192 (PMC12159973; doi:10.1021/acs.jctc.5c00192)
Supplement: Supplementary file 1 [file ct5c00192_si_001.pdf]

Supporting Information

Probing limitations of co-alchemical charge changes in free-energy calculations

Nadine Grundschober and Dražen Petrov*

E-mail: drazen.petrov@boku.ac.at

BOKU University

Institute of Molecular Modeling and Simulation

Department of Natural Sciences and Sustainable Resources

Muthgasse 18, 1190 Vienna, Austria

Contents

Simulated systems S2

Perturbation using a charged co-alchemical ion S4

Systems neutralized with free counterions S4

Umbrella sampling S6

The role of the box size S8

Copies S10

Neutral compound - Asparagine side chain analog S10

Initial coordinates of each of the ions S11

Free energies copies S13

Charge distribution S17

Simulated systems

Table S1. Summary of simulated systems.

Charge	Cl^- -> Cl^0 perturbation	Co-perturbed ion perturbation	Position of the co-ion	Number of free counterions
Na^+	Cl^- -> Cl^0	Cl^0 -> Cl^-	(1.99, 0, 0)	0
Na^{2+}	Cl^- -> Cl^0	Cl^0 -> Cl^-	(1.99, 0, 0)	1
Na^{4+}	Cl^- -> Cl^0	Cl^0 -> Cl^-	(1.99, 0, 0)	3
Na^{7+}	Cl^- -> Cl^0	Cl^0 -> Cl^-	(1.99, 0, 0)	6
Na^{2+}	Cl^- -> Cl^0	Cl^{1-} -> Cl^{2-}	(1.99, 0, 0)	0
Na^{4+}	Cl^- -> Cl^0	Cl^{3-} -> Cl^{4-}	(1.99, 0, 0)	0
Na^{7+}	Cl^- -> Cl^0	Cl^{6-} -> Cl^{7-}	(1.99, 0, 0)	0
Na^+	Cl^- -> Cl^0	Cl^0 -> Cl^-	(1.99, 1.99, 1.99)	0
Na^{2+}	Cl^- -> Cl^0	Cl^0 -> Cl^-	(1.99, 1.99, 1.99)	1
Na^{4+}	Cl^- -> Cl^0	Cl^0 -> Cl^-	(1.99, 1.99, 1.99)	3
Na^{7+}	Cl^- -> Cl^0	Cl^0 -> Cl^-	(1.99, 1.99, 1.99)	6
Na^{2+}	Cl^- -> Cl^0	Cl^{1-} -> Cl^{2-}	(1.99, 1.99, 1.99)	0
Na^{4+}	Cl^- -> Cl^0	Cl^{3-} -> Cl^{4-}	(1.99, 1.99, 1.99)	0
Na^{7+}	Cl^- -> Cl^0	Cl^{6-} -> Cl^{7-}	(1.99, 1.99, 1.99)	0
Na^+	Cl^- -> Cl^0	Cl^0 -> Cl^-	(1.15, 1.15, 1.15)	0
Na^{2+}	Cl^- -> Cl^0	Cl^0 -> Cl^-	(1.15, 1.15, 1.15)	1
Na^{4+}	Cl^- -> Cl^0	Cl^0 -> Cl^-	(1.15, 1.15, 1.15)	3
Na^{7+}	Cl^- -> Cl^0	Cl^0 -> Cl^-	(1.15, 1.15, 1.15)	6
Na^{2+}	Cl^- -> Cl^0	Cl^{1-} -> Cl^{2-}	(1.15, 1.15, 1.15)	0
Na^{4+}	Cl^- -> Cl^0	Cl^{3-} -> Cl^{4-}	(1.15, 1.15, 1.15)	0
Na^{7+}	Cl^- -> Cl^0	Cl^{6-} -> Cl^{7-}	(1.15, 1.15, 1.15)	0

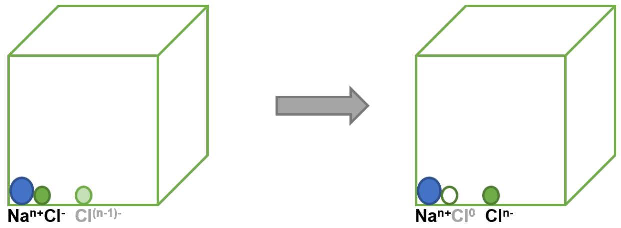

Figure S1. Setup of the co-alchemical perturbation along the x-coordinate consisting of a fixed charged Na^{n+} and a Cl^- acting as a *complex* and the co-alchemically perturbed Cl-ion starting with a negative charge of (n-1). The single charge of the Cl^- was then co-alchemically perturbed to the $Cl^{(n-1)-}$ placed at $(\frac{a}{2})$ of the box length resulting in Cl^{n-}.

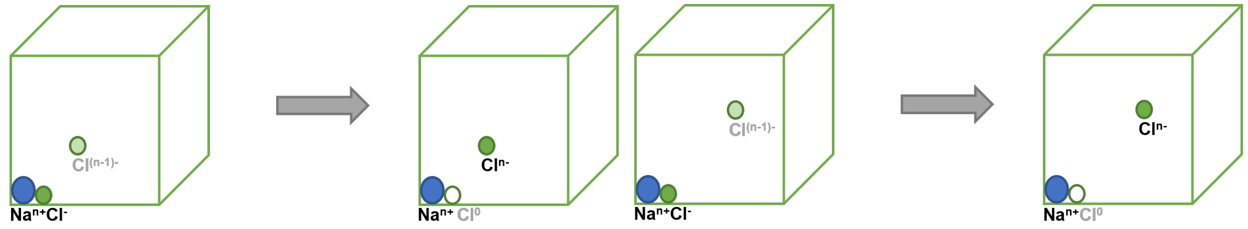

(a) (b)

Figure S2. Setup of the co-alchemical perturbation along the xyz-coordinate consisting of a fixed charged Na^{n+} and a Cl^- acting as a *complex* and the co-alchemically perturbed Cl-ion starting with a negative charge of (n-1). The single charge of the Cl^- was then co-alchemically perturbed to the $Cl^{(n-1)-}$ placed at (a) $(\frac{a}{2})$ (b) $(\frac{d}{2})$ of the box resulting in Cl^{n-}.

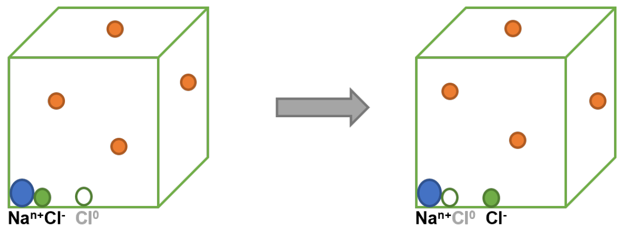

Figure S3. Example setup of the co-alchemical perturbation in x-direction with a fixed charged Na^{n+} and a Cl^- as *complex* and the neutral co-alchemically perturbed Cl-ion. The single charge of the Cl^- was then co-alchemically perturbed to the Cl^0 placed at $(\frac{a}{2})$ of the box length. To ensure a neutral system free counterions (Cl^-, showed in orange) were added.

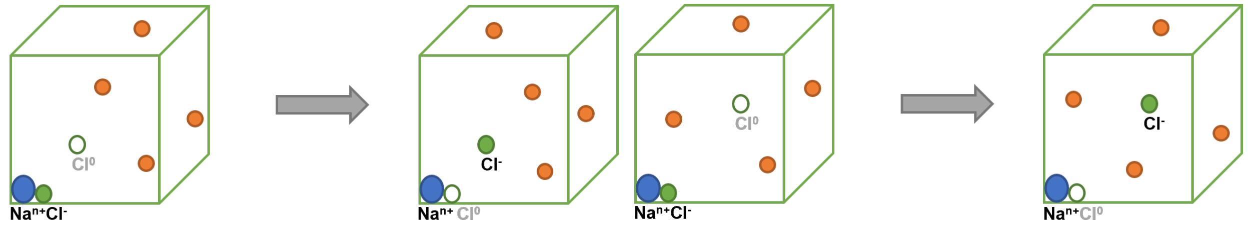

(a) (b)

Figure S4. Example setup of the co-alchemical perturbation in xyz-direction applying with a fixed charged $Na^{n+}Cl^-$ as *complex* and the neutral co-alchemically perturbed Cl-ion. The single charge of the Cl^- was then co-alchemically perturbed to the Cl^0 placed at (a) $(\frac{a}{2})$ or (b) $(\frac{d}{2})$ of the box resulting in Cl^{n-}. To ensure a neutral system free counterions (Cl^-, showed in orange) were added.

Perturbation using a charged co-alchemical ion

Table S2. Results of the effect of the direction and the distance of the co-alchemical ion by migrating the charge from the bound Cl^- to the co-alchemical ion using ($\frac{a}{2}$) in x- and xyz-direction and ($\frac{d}{2}$) in xyz-direction.

Charge	ΔG_bar [kJ mol^{-1}]	Error_bar [kJ mol^{-1}]	Error_BS [kJ mol^{-1}]	ΔG_BS [kJ mol^{-1}]	Boxsize [nm]	Free counterions	Direction	Distance [nm]
Na^{+}	35.7	0.0	0.1	35.4	4	0	x	2.0
Na^{+}	35.6	0.0	0.2	35.5	4	0	xyz	2.0
Na^{+}	36.0	0.1	0.2	35.8	4	0	xyz	3.5
Na^{2+}	-670.5	1.0	2.7	-669.0	4	0	x	2.0
Na^{2+}	-671.7	1.4	2.9	-672.8	4	0	xyz	2.0
Na^{2+}	-669.9	0.7	1.2	-669.6	4	0	xyz	3.5
Na^{4+}	-1464.0	3.3	24.4	-1816.8	4	0	x	2.0
Na^{4+}	-1468.3	5.7	27.1	-1852.5	4	0	xyz	2.0
Na^{4+}	-1449.2	4.8	27.9	-1819.9	4	0	xyz	3.5
Na^{7+}	-1472.8	7.5	297.3	-2839.5	4	0	x	2.0
Na^{7+}	-1480.0	12.8	451.2	-3314.6	4	0	xyz	2.0
Na^{7+}	-1449.5	10.3	350.7	-3005.1	4	0	xyz	3.5

Table S3. Summary of the free energies of the charge perturbation in different direction without counterions.

Charge	x-direction (distance: $\frac{a}{2}$)	xyz-direction (distance: $\frac{a}{2}$)	xyz-direction (distance: $\frac{d}{2}$)
Na^{+}	35.7 ± 0 kJ mol^{-1}	35.6 ± 0 kJ mol^{-1}	36.0 ± 0.1 kJ mol^{-1}
Na^{2+}	-670.5 ± 1.0 kJ mol^{-1}	-671.7 ± 1.4 kJ mol^{-1}	-669.9 ± 0.7 kJ mol^{-1}
Na^{4+}	-1464.0 ± 3.3 kJ mol^{-1}	-1468.3 ± 5.7 kJ mol^{-1}	-1449.2 ± 4.8 kJ mol^{-1}
Na^{7+}	-1472.8 ± 7.5 kJ mol^{-1}	-1480.0 ± 12.8 kJ mol^{-1}	-1449.5 ± 10.3 kJ mol^{-1}

Systems neutralized with free counterions

Table S4. Results of the perturbed Cl^- to the same distance in x- and xyz-direction and to ($\frac{d}{2}$) in xyz-direction using the setups with free counterions.

Charge	ΔG_bar [kJ mol^{-1}]	Error_bar [kJ mol^{-1}]	Error_BS [kJ mol^{-1}]	ΔG_BS [kJ mol^{-1}]	Boxsize [nm]	Free counterions	Direction	Distance [nm]
Na^{2+}	117.7	0.2	0.1	117.7	4	1	x	2.0
Na^{2+}	118.2	0.2	0.1	118.1	4	1	xyz	2.0
Na^{2+}	118.3	0.1	0.2	118.6	4	1	xyz	3.5
Na^{4+}	332.9	0.5	0.1	332.7	4	3	x	2.0
Na^{4+}	332.5	0.5	0.1	332.8	4	3	xyz	2.0
Na^{4+}	329.8	2.0	0.1	329.5	4	3	xyz	3.5
Na^{7+}	778.5	1.9	0.1	778.5	4	6	x	2.0
Na^{7+}	792.0	0.9	0.1	792.1	4	6	xyz	2.0
Na^{7+}	798.2	1.4	0.1	798.0	4	6	xyz	3.5

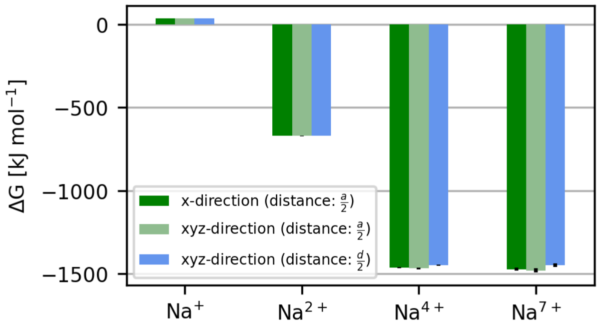

Figure S5. Free energy differences of the displacement of the co-alchemical ion in x- and xyz-direction. The charge of the Cl^- was migrated to the co-perturbed ion that was charged to the same amount as the charge of the Na-ion using the same distance in x- and xyz-direction or half of the diagonal length of the box. An increase of the charge on the Na-ion leads to a decrease of the free energy differences. Starting with a charge of Na^{4+} the error estimates are much higher then the free energy differences, which makes an interpretation of the impact of the distance or direction difficult.

Table S5. Summary of the free energies of the charge perturbation in different direction with free counterions.

Charge	x-direction (distance: $\frac{a}{2}$)	xyz-direction (distance: $\frac{a}{2}$)	xyz-direction (distance: $\frac{d}{2}$)
Na^{2+}	117.7 ± 0.2 kJ mol^{-1}	118.2 ± 0.2 kJ mol^{-1}	118.3 ± 0.1 kJ mol^{-1}
Na^{4+}	332.9 ± 0.5 kJ mol^{-1}	332.5 ± 0.5 kJ mol^{-1}	329.8 ± 2.0 kJ mol^{-1}
Na^{7+}	778.5 ± 1.9 kJ mol^{-1}	792.0 ± 0.9 kJ mol^{-1}	798.2 ± 1.4 kJ mol^{-1}

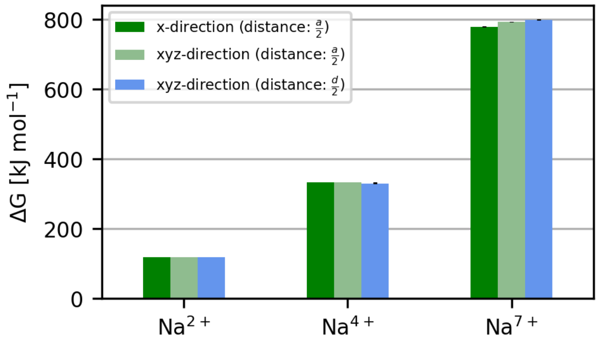

(a)

Figure S6. Free energy differences of the perturbed charge of the ion to the same distance in x- or xyz-direction or to $(\frac{d}{2})$ using Na^{2+}/ Na^{4+}/$Na^{7+}Cl^-$ *complex* with free counterions to keep the system neutralized.

Umbrella sampling

The Cl-anion was pulled along the x- and xyz-reaction coordinate away from the Na-cation, where the xyz refers to the space diagonal of the simulation box. Furthermore, two sets of charges were applied (Na^+ and Na^{4+}). Each of the umbrella windows was simulated for 20 ns. The intermediate states were covered by a series of windows along the respective reaction coordinate using a force constant of 1,000 $kJ\,mol^{-1}\,nm^{-2}$. Afterwards, the windows were combined by weighted histogram analysis to determine the potentials of mean force (PMFs) along the reaction coordinate starting from 0.5 nm.

Table S6. PMFs of Na^+ and Na^{4+}

**Charge**	**Box size [nm]**	**Direction**	
		**x**	**xyz**
Na^+	4	4.9 kJ mol^{-1}	4.2 kJ mol^{-1}
Na^+	8	4.5 kJ mol^{-1}	4.4 kJ mol^{-1}
Na^{4+}	4	9.9 kJ mol^{-1}	11.2 kJ mol^{-1}
Na^{4+}	8	14.3 kJ mol^{-1}	14.6 kJ mol^{-1}

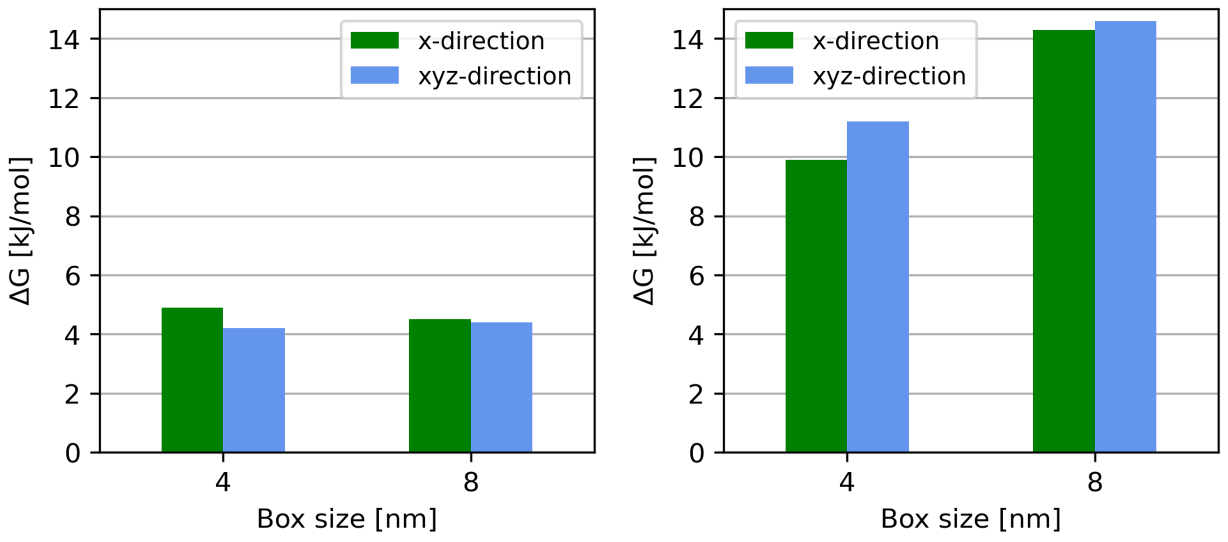

(a) (b)

Figure S7. The graph shows a comparison of different pulling setups and two different charges of the Na-ion. a) For the Na^+ setup neither the box size nor the direction has an impact on the free energies. b) Using a charge of Na^{4+} the direction has no significant influence on the PMF values but an increase of the ΔGs can be observed between the 4 and 8 nm box in x-direction.

The umbrella sampling of a single charged Na-ion in x- and xyz-direction in two different box sizes resulted in similar ΔGs (Table S6). The results show that neither the direction nor the distance of the ion representing the *unbound* state did affect the free energies using a charge of Na^+ (Figure S7a). For a charge of Na^{4+} the PMF is similar across different directions but the ΔGs are increasing between a box size of 4 and 8 nm pulling the co-alchemical ion in x-direction (Figure S7b).

The role of the box size

Table S7. The charge of the Cl-ion of the $Na^{2+}Cl^{-}$ and the $Na^{4+}Cl^{-}$ *complex* was migrated to $(\frac{d}{2})$ of the box length side in a 4, 6, 8 and 10 nm box. For systems containing small charges the box size does not matter, whereas, the systems with a charge of Na^{4+} the calculated free energies are strongly dependent on the box size.

Charge	ΔG_bar [kJ mol^{-1}]	Error_bar [kJ mol^{-1}]	Error_BS [kJ mol^{-1}]	ΔG_BS [kJ mol^{-1}]	Boxsize [nm]	Free counterions	Direction	Distance [nm]
Na^{2+}	118.3	0.1	0.2	118.6	4	1	xyz	3.5
Na^{2+}	118.8	0.1	0.2	118.7	6	1	xyz	5.2
Na^{2+}	119.0	0.1	0.1	119.0	8	1	xyz	6.9
Na^{2+}	119.3	0.2	0.2	119.3	10	1	xyz	8.7
Na^{4+}	329.8	2.0	0.1	329.5	4	3	xyz	3.5
Na^{4+}	335.5	0.7	0.1	335.6	6	3	xyz	5.2
Na^{4+}	337.3	0.7	0.1	337.3	8	3	xyz	6.9
Na^{4+}	338.9	0.3	0.1	339.3	10	3	xyz	8.7
Na^{4+}	338.9	0.4	0.1	338.4	12	3	xyz	10.4
Na^{4+}	340.2	0.2	0.1	340.3	14	3	xyz	12.1

Table S8. The charge of the Na^{+}-ion of the ($Cl^{4-}Na^{+}$) *complex* was perturbed in xyz-direction. This suggests a dependency of the free energies on the box size.

Charge	ΔG_bar [kJ mol^{-1}]	Error_bar [kJ mol^{-1}]	Error_BS [kJ mol^{-1}]	ΔG_BS [kJ mol^{-1}]	Boxsize [nm]	Free counterions	Direction	Distance [nm]
Cl^{2-}	70.9	2.5	0.2	71.0	4	1	xyz	3.5
Cl^{2-}	80.3	0.5	0.2	80.3	6	1	xyz	5.2
Cl^{2-}	78.9	3.0	0.2	79.0	8	1	xyz	6.9
Cl^{2-}	85.0	0.5	0.2	84.8	10	1	xyz	8.7
Cl^{2-}	81.6	1.9	0.2	81.4	12	1	xyz	10.4
Cl^{2-}	85.2	0.6	0.2	85.0	14	1	xyz	12.1
Cl^{4-}	243.9	7.9	0.4	244.1	4	3	xyz	3.5
Cl^{4-}	251.6	7.5	0.3	251.9	6	3	xyz	5.2
Cl^{4-}	285.4	11.7	0.2	285.1	8	3	xyz	6.9
Cl^{4-}	316.9	8.7	0.2	316.7	10	3	xyz	8.7
Cl^{4-}	351.1	3.1	0.2	351.2	12	3	xyz	10.4
Cl^{4-}	344.6	6.2	0.2	344.5	14	3	xyz	12.1

Effect of salt concentration on the free energy box-size dependence

Table S9. The charge of the Cl-ion of the $Na^{4+}Cl^{-}$ *complex* was migrated to $(\frac{d}{2})$ of the box length side in a 4, 6, 8, 10, 12 and 14 nm box. The charge of the Na^{+}-ion of the $(Cl^{2-}Na^{+})$ and of the $(Cl^{4-}Na^{+})$ *complex* was perturbed in xyz-direction.

Charge	ΔG_bar [kJ mol^{-1}]	Error_bar [kJ mol^{-1}]	Error_BS [kJ mol^{-1}]	ΔG_BS [kJ mol^{-1}]	Boxsize [nm]	Direction	Distance [nm]	Salt concentration [M]
Na^{4+}	343.3	1.8	0.2	343.4	4	xyz	3.5	0.15
Na^{4+}	330.7	3.3	0.2	330.8	6	xyz	5.2	0.15
Na^{4+}	328.5	3.1	0.2	328.8	8	xyz	6.9	0.15
Na^{4+}	332.0	1.4	0.2	332.0	10	xyz	8.7	0.15
Na^{4+}	330.2	1.5	0.2	330.2	12	xyz	10.4	0.15
Na^{4+}	331.2	1.9	0.2	331.6	14	xyz	12.1	0.15
Cl^{2-}	44.2	0.8	0.2	44.1	4	xyz	3.5	0.15
Cl^{2-}	38.8	1.4	0.2	38.6	6	xyz	5.2	0.15
Cl^{2-}	42.6	2.9	0.2	42.8	8	xyz	6.9	0.15
Cl^{2-}	39.2	1.1	0.2	39.2	10	xyz	8.7	0.15
Cl^{2-}	42.9	2.0	0.2	42.7	12	xyz	10.4	0.15
Cl^{2-}	37.9	1.3	0.2	37.9	14	xyz	12.1	0.15
Cl^{4-}	138.1	2.5	0.2	138.1	4	xyz	3.5	0.15
Cl^{4-}	153.1	5.1	0.2	153.3	6	xyz	5.2	0.15
Cl^{4-}	147.4	3.4	0.2	146.9	8	xyz	6.9	0.15
Cl^{4-}	162.9	7.7	0.2	162.6	10	xyz	8.7	0.15
Cl^{4-}	156.5	10.2	0.2	156.8	12	xyz	10.4	0.15
Cl^{4-}	154.7	2.7	0.2	154.6	14	xyz	12.1	0.15

Protein-ligand system

Table S10. The charge of the protonated amino group of the ligand-290 was perturbed onto a co-alchemical ion, which was kept at a distance of a half of the spatial diagonal of the box from the amino group. Two setups, without and with a salt concentration 0.15 M NaCl was tested. The results suggest a box-size dependence, with the effect being more pronounced in simulations that do not include salt.

ΔG_bar [kJ mol^{-1}]	Error_bar [kJ mol^{-1}]	Error_BS [kJ mol^{-1}]	ΔG_BS [kJ mol^{-1}]	Boxsize [nm]	Free counterions	Direction	Distance [nm]	Salt concentration [M]
-101.8	3.3	0.1	-101.7	6	4	xyz	5.2	0
-80.2	3.6	0.1	-80.0	8	4	xyz	6.9	0
-99.6	4.3	0.1	-99.6	10	4	xyz	8.7	0
-77.3	5.6	0.1	-77.4	12	4	xyz	10.4	0
-74.2	5.5	0.1	-74.4	14	4	xyz	12.1	0
-97.5	0.9	0.1	-97.6	6		xyz	5.2	0.15
-79.6	3.0	0.1	-76.0	8		xyz	6.9	0.15
-97.3	2.8	0.1	-97.4	10		xyz	8.7	0.15
-84.4	1.2	0.1	-84.2	12		xyz	10.4	0.15
-87.0	3.4	0.1	-86.8	14		xyz	12.1	0.15

Copies

Neutral compound - Asparagine side chain analog

To test for potential pitfalls of the multi-copy approach, a setup consisting of the Asparagine side chain analog, a neutral compound, perturbed into a neutral non-interacting dummy compound was simulated. One copy was embedded in a 4 nm box and four copies were placed in a 6.35 nm box (the same molar concentration). Most of the simulation parameters were kept similar, except that the MD simulations were performed for 20 ns per lambda point.

Table S11. Placing a single copy or four copies of the Asparagine side chain analog in a box results in a similar total free energy per copy compared to the single copy.

Label	ΔG_bar [kJ mol^{-1}]	Error_bar [kJ mol^{-1}]	Error_BS [kJ mol^{-1}]	ΔG_BS [kJ mol^{-1}]	Boxsize [nm]
individual copy$_1$	243.0	0.1	0.3	243.1	6.35
individual copy$_2$	243.1	0.1	0.3	243.3	6.35
individual copy$_3$	243.1	0.1	0.3	243.0	6.35
individual copy$_4$	243.1	0.2	0.3	243.1	6.35
multi copy	243.2			243.2	6.35
disentangled	243.2	0.0	0.0	243.1	6.35
single	243.2	0.1	0.1	243.3	6.35

This results show that placing more copies in a box leads to similar results also with applying the disentanglement for a neutral compound.

Initial coordinates of each of the ions

Table S12. Initial coordinates of the ions of four copies.

(a) Initial coordinates of the ions of the four copies placed in the 4 and 6.35 nm box (Figure 9a).

**Copy**	**Coordinates**
Na_1	(0, 0, 0)
$Cl_1^{complex}$	(0.27, 0, 0)
$Cl_1^{co-alch.}$	(1.99, 0, 0)
Na_2	(1.99, 1.99, 0)
$Cl_2^{complex}$	(2.27, 1.99, 0)
$Cl_2^{co-alch.}$	(0, 1.99, 0)
Na_3	(1.99, 0, 1.99)
$Cl_3^{complex}$	(2.27, 0, 1.99)
$Cl_3^{co-alch.}$	(0, 0, 1.99)
Na_4	(0, 1.99, 1.99)
$Cl_4^{complex}$	(0.27, 1.99, 1.99)
$Cl_4^{co-alch.}$	(1.99, 1.99, 1.99)

(b) Initial coordinates of the ions of the four copies placed differently oriented in a 6.35 nm box (Figure 9b).

**Copy**	**Coordinates**
Na_1	(0, 0, 0)
$Cl_1^{complex}$	(0.27, 0, 0)
$Cl_1^{co-alch.}$	(3.18, 0, 0)
Na_2	(0, 3.18, 0)
$Cl_2^{complex}$	(0, 2.9, 0)
$Cl_2^{co-alch.}$	(3.18, 3.18, 0)
Na_3	(3.18, 0, 0)
$Cl_3^{complex}$	(2.9, 0, 3.18)
$Cl_3^{co-alch.}$	(0, 0, 3.18)
Na_4	(3.18, 3.18, 3.18)
$Cl_4^{complex}$	(3.18, 3.18, 3.18)
$Cl_4^{co-alch.}$	(0, 3.18, 3.18)

Table S13. Initial coordinates of the ions of three and eight copies.

(a) Initial coordinates of the ions of the three copies differently oriented (Figure 9d).

**Copy**	**Coordinates**
Na_1	(2.99, 0, 0)
$Cl_1^{complex}$	(2.99, 0.27, 0)
$Cl_1^{co-alch.}$	(2.99, 2.99, 0)
Na_2	(0, 2.99, 0)
$Cl_2^{complex}$	(0, 2.99, 0.27)
$Cl_2^{co-alch.}$	(0, 2.99, 2.99)
Na_3	(0, 0, 2.99)
$Cl_3^{complex}$	(0.27, 0, 2.99)
$Cl_3^{co-alch.}$	(2.99, 0, 2.99)

(b) Initial coordinates of the ions of the eight copies in a 8 nm box (Figure 9c).

**Copy**	**Coordinates**
Na_1	(0, 0, 0)
$Cl_1^{complex}$	(0.27, 0, 0)
$Cl_1^{co-alch.}$	(2.27, 1.99, 1.99)
Na_2	(3.99, 3.99, 0)
$Cl_2^{complex}$	(4.27, 3.99, 0)
$Cl_2^{co-alch.}$	(6.27, 5.99, 1.99)
Na_3	(3.99, 0, 3.99)
$Cl_3^{complex}$	(4.267, 0, 3.99)
$Cl_3^{co-alch.}$	(6.27, 1.99, 5.99)
Na_4	(0, 3.99, 3.99)
$Cl_4^{complex}$	(0.27, 3.99, 3.99)
$Cl_4^{co-alch.}$	(2.27, 5.99, 3.9)9
Na_5	(3.99, 0, 0)
$Cl_5^{complex}$	(4.27, 0, 0)
$Cl_5^{co-alch.}$	(6.27, 1.99, 1.99)
Na_6	(0, 3.99, 0)
$Cl_6^{complex}$	(0.27, 3.99, 0)
$Cl_6^{co-alch.}$	(2.27, 5.99, 1.99)
Na_7	(0, 0, 3.99)
$Cl_7^{complex}$	(0.27, 0, 3.99)
$Cl_7^{co-alch.}$	(2.27, 1.99, 5.99)
Na_8	(3.99, 3.99, 3.99)
$Cl_8^{complex}$	(4.27, 3.99, 3.99)
$Cl_8^{co-alch.}$	(6.27, 5.99, 5.99)

Free energies copies

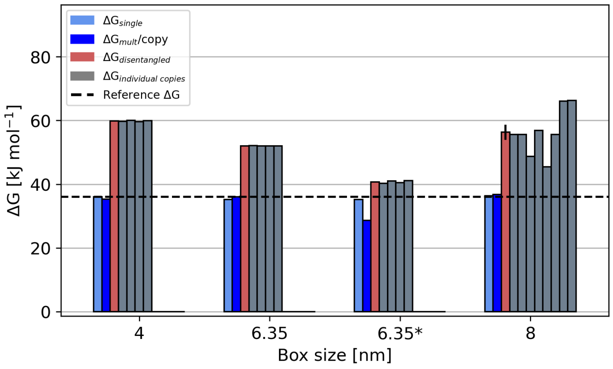

Figure S8. The graph shows a comparison of different ΔGs representing: the ΔG of a single copy in the box in light blue (ΔG_{single}, data from Fig 5), the total ΔG of multi-copy perturbation normalized by the number of copies in dark blue (ΔG_{mult}/copy), the average ΔG of the disentangled copies in the box in red ($\Delta G_{disentangled}$) and the disentangled ΔGs of the individual copies using the setup Na^{+}. The free energies are compared to the reference ΔG in the biggest simulated box. Interestingly, the disentanglement of the individual copies results in higher free energies compared to a single copy or the total free energy per copy. In the boxes marked with a * the copies were oriented differently, which resulted in ΔGs comparable to the reference ΔG_{single}.

Table S14. Free energies of the individual copies in different box sizes using a charge of Na^{+} and Na^{4+}.

**Na^{+}**					
Label	ΔG_bar [kJ mol^{-1}]	Error_bar [kJ mol^{-1}]	Error_BS [kJ mol^{-1}]	ΔG_BS [kJ mol^{-1}]	Boxsize [nm]
individual copy$_1$	59.7	0.1	0.3	59.7	4
individual copy$_2$	60.0	0.1	0.3	59.5	4
individual copy$_3$	59.6	0.1	0.3	59.8	4
individual copy$_4$	59.9	0.1	0.3	59.7	4
multi copy	35.3			35.1	4
disentangled	59.8	0.1	0.1	59.7	4
single	36.0	0.1	0.1	36.3	4

Label	ΔG_bar [kJ mol^{-1}]	Error_bar [kJ mol^{-1}]	Error_BS [kJ mol^{-1}]	ΔG_BS [kJ mol^{-1}]	Boxsize [nm]
individual copy$_1$	52.1	0.1	0.3	51.9	6.35
individual copy$_2$	52.0	0.1	0.3	52.0	6.35
individual copy$_3$	52.0	0.1	0.3	52.1	6.35
individual copy$_4$	52.0	0.1	0.3	51.6	6.35
multi copy	36.0			36.1	6.35
disentangled	52.0	0.0	0.1	51.9	6.35
single	35.2	0.1	0.1	36.3	6.35
individual copy$_1$	40.3	0.1	0.3	40.1	6.35*
individual copy$_2$	41.0	0.1	0.3	41.0	6.35*
individual copy$_3$	40.5	0.2	0.3	39.6	6.35*
individual copy$_4$	41.1	0.2	0.3	41.0	6.35*
multi copy	28.6			36.1	6.35*
disentangled	40.7	0.2	0.3	40.5	6.35*
single	35.2	0.1	0.1	36.3	6.35*
individual copy$_1$	55.6	0.1	0.3	55.4	8
individual copy$_2$	55.6	0.1	0.3	55.7	8
individual copy$_3$	48.7	0.2	0.3	65.4	8
individual copy$_4$	56.9	0.1	0.3	48.3	8
individual copy$_5$	45.5	0.2	0.3	57.0	8
individual copy$_6$	55.6	0.1	0.3	45.1	8
individual copy$_7$	66.1	0.2	0.3	65.9	8
individual copy$_8$	66.3	0.2	0.3	66.1	8
multi copy	36.8			35.4	8
disentangled	56.3	2.4	2.7	57.4	8
single	36.4	0.1	0.1	36.3	8

Label	ΔG_bar [kJ mol^{-1}]	Error_bar [kJ mol^{-1}]	Error_BS [kJ mol^{-1}]	ΔG_BS [kJ mol^{-1}]	Boxsize [nm]
**Na^{4+}**					
individual copy$_1$	355.4	1.2	0.2	355.3	4
individual copy$_2$	355.5	0.4	0.3	355.0	4
individual copy$_3$	354.4	0.6	0.2	354.6	4
individual copy$_4$	355.7	0.9	0.2	355.5	4
multi copy	330.4			331.0	4
disentangled	355.2	0.2	0.2	355.1	4
single	329.8	2.0	0.7	337.3	4
individual copy$_1$	340.3	0.7	0.3	334.7	6
individual copy$_2$	340.8	0.7	0.2	340.8	6
individual copy$_3$	338.9	0.4	0.2	340.6	6
multi copy	333.7			332.6	6
disentangled	340.0	0.5	1.6	338.7	6
single	335.5	0.7	0.7	337.3	6
individual copy$_1$	349.7	0.3	0.3	349.7	6.35
individual copy$_2$	349.4	0.6	0.6	349.4	6.35
individual copy$_3$	349.6	0.7	0.7	349.3	6.35
individual copy$_4$	343.9	0.4	0.4	343.6	6.35
multi copy	331.9			331.7	6.35
disentangled	348.1	1.2	1.3	348.0	6.35
single	336.7	0.5	0.7	337.3	6.35
individual copy$_1$	337.5	1.2	0.3	336.8	6.35*
individual copy$_2$	339.0	0.6	0.3	338.6	6.35*
individual copy$_3$	337.4	0.8	0.2	337.3	6.35*
individual copy$_4$	339.5	0.4	0.3	339.6	6.35*

Label	ΔG_bar [kJ mol^{-1}]	Error_bar [kJ mol^{-1}]	Error_BS [kJ mol^{-1}]	ΔG_BS [kJ mol^{-1}]	Boxsize [nm]
multi copy	333.6			334.1	6.35*
disentangled	338.3	0.5	0.5	338.1	6.35*
single	336.7	0.5	0.7	337.3	6.35*
individual copy$_1$	354.6	0.1	0.3	354.3	8
individual copy$_2$	354.3	0.5	0.3	354.9	8
individual copy$_3$	359.5	0.3	0.3	359.5	8
individual copy$_4$	344.1	1.2	0.3	343.6	8
individual copy$_5$	354.0	0.5	0.3	353.6	8
individual copy$_6$	345.0	0.4	0.3	344.6	8
individual copy$_7$	364.7	0.5	0.3	364.4	8
individual copy$_8$	356.4	0.6	0.3	356.7	8
multi copy	332.9			331.7	8
disentangled	354.1	2.3	2.3	353.9	8
single	337.3	0.7	0.7	337.3	8

The disentanglement of the individual copies resulted in much higher ΔGs compared to a single copy. However, dividing the total free energy by the amount of copies resulted in similar or lower free energies compared to a single copy (Figure 8). The four copies in the 4 nm box using a charge of Na^+ resulted in a free energy of approximately 59 kJ mol $^{-1}$ of each copy, whereas, four copies in a 6.35 nm box result in lower energies per copy (52 ± 0.1 kJ mol $^{-1}$, Figure S8). Nevertheless, the free energies of the individual copies are higher compared to the setup with only one copy in the box, which showed a ΔG of 36.0 ± 0.1 kJ mol $^{-1}$ (also comparable to the reference ΔG). Dividing the total ΔG_{mult} by the number of copies, the same free energies as for a single copy are achieved. The eight copies in the 8 nm box show a higher deviation of the free-energies of the individual copies.

Distributed co-alchemical charge perturbation

To avoid a choice of direction to which the co-perturbed ion is displaced, the co-perturbed charge was uniformly distributed around the 2-ion *complex* in this setup. To achieve this, the charge of -1.2 was divided across six $Cl^{0.2-}$ which were placed ($\frac{a}{4}$) away from the *complex* in x-, y- and z-direction, as displayed in Table S15. This setup was applied to the $Na^{4.2+}$ $Cl^{1.2-}$ *complex* only, and was performed in five different box sizes.

Table S15. xyz-coordinates of the co-perturbed $Cl^{1.2-}$ ions in the box, where a stands for the length of the box. The six $Cl^{1.2-}$ ions were uniformly distributed around the $Na^{4.2+}$ $Cl^{1.2-}$ *complex* to cover all directions.

**$Cl^{1.2-}$ Ion**	**Coordinates**		
	**x**	**y**	**z**
1	$\frac{a}{4}$	0	0
2	$\frac{3a}{4}$	0	0
3	0	$\frac{a}{4}$	0
4	0	$\frac{3a}{4}$	0
5	0	0	$\frac{a}{4}$
6	0	0	$\frac{3a}{4}$

Also with this setup free energies dependent on the box size were obtained (Figure S9). Similarly to the *simple* setup with one co-alchemical ion, free energies independent of the box size can be achieved using a bigger box size, for example, a box size of 10 nm (see Table S16). Similar free-energy differences of about 10 kJ mol $^{-1}$ between a 4 nm box and a 10 nm box are calculated, as compared to the setup with a similar charge and a single co-alchemical ion (Figure 4). Note that the calculated free energy is much higher, due to overall different perturbation applied, including a different charge of $Cl^{1.2-}$. Since setting up a system with charge distribution is harder, and no improvement is achieved, using a more standardized scheme is preferred.

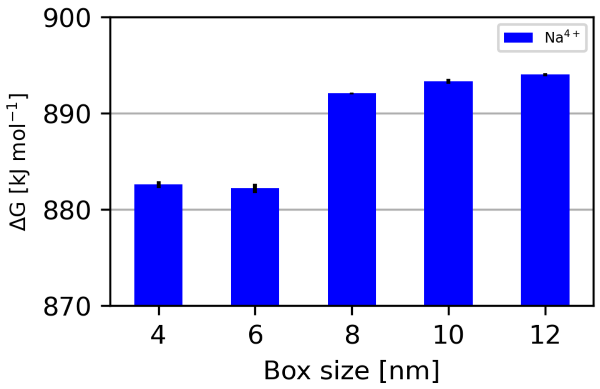

Figure S9. Results of the $Na^{4+}Cl^{1.2-}$ setup in which the migrating charge was distributed across six co-perturbed ions. With an increase of the box size the free energies are increasing, showing a dependency of the free energies on the box size. Free energy independent of the box size can be achieved using a box size of at least 10 nm.

Table S16. Results of the $Na^{4+}Cl^{1.2-}$ setup in which the migrating charge was distributed across six co-perturbed ions. With an increase of the box size the free energies are increasing, showing a dependency of the free energies on the box size. Free energy independent of the box size can be achieved using a box size of at least 10 nm.

Boxsize [nm]	ΔG_bar [kJ mol^{-1}]	Error_bar [kJ mol^{-1}]	Error_BS [kJ mol^{-1}]	ΔG_BS [kJ mol^{-1}]	Free counterions
4	882.6	0.3	0.1	883.4	3
6	882.2	0.5	0.1	886.1	3
8	892.1	0.1	0.1	890.8	3
10	893.3	0.2	0.1	893.5	3
12	894.0	0.2	0.1	893.7	3
